# Edible Lepidoptera in Mexico: Geographic distribution, ethnicity, economic and nutritional importance for rural people

**DOI:** 10.1186/1746-4269-7-2

**Published:** 2011-01-06

**Authors:** Julieta Ramos-Elorduy, José MP Moreno, Adolfo I Vázquez, Ivonne Landero, Héctor Oliva-Rivera, Víctor HM Camacho

**Affiliations:** 1 Instituto de Biología, UNAM, Apartado Postal 70-153, 04510, México DF, México; 2 Facultad de Ciencias Biológicas y Agropecuarias UV, Córdoba Veracruz, México

## Abstract

In this paper, we reported the butterflies and moths that are consumed in Mexico. We identified 67 species of Lepidoptera that are eaten principally in their larval stage in 17 states of Mexico. These species belong to 16 families: Arctiidae, Bombycidae, Castniidae, Cossidae, Geometridae, Hepialidae, Hesperiidae, Lasiocampidae, Noctuidae, Nymphalidae, Papilionidae, Pieridae, Pyralidae, Saturniidae, Sesiidae, and Sphingidae.

Saturniidae, Pieridae, Noctuidae and Nymphalidae were the more species consumed with 16, 11, 9, and 8 species, respectively.

The genera with the largest numbers of species were: *Phassus*, *Phoebis*, *Hylesia *and *Spodoptera*, with three species.

Their local distribution, corresponding to each state of Mexico, is also presented.

## Background

Lepidoptera is one of the richest Insecta orders. Their larvae serve as food for many ethnic groups around the world [1,2]; they are often prepared charcoaled in salty water or, in some cases, fried or mixed with other food [3]. Also contribute a great amount of energy and protein to indigenous diet [4]. In general, this reflects their availability. In the forests of the Central African Republic, some species are so abundant, that when they are in the last larval stage, their excrement fall sounding like heavy raindrops, and two months later, the soil becomes white due to the mycelium that develops [Ramos-Elorduy J, Personal observations, 1990].

The inhabitants make good use of them, storing and selling. This help the people to obtain income that is necessary in a subsistence economy. Ancient Mexicans were traded larvae of *Pantherodes pardalaria *and *Aegiale hesperiaris *[5]. Peasants know very well when and where is the biggest and tasty larval stage. People even make long journeys to obtain them; however, because of an over-exploitation, as in Zambia happened, establish a law to enforce a closed season, to prevent extinction of *Gynanisa maja *and *Gonimbrasia belina *named "mumpa" [6], exploiting it in a rational way to balance preservation and exploitation [7].

The use of insects as food by the different ethnia of Mexico is a very complete study at Mexico that achieve 549 species [8]. We have documented 14 orders of the Insecta Class, including Lepidoptera.

A study of the edible species of Lepidoptera in Mexico has not yet been accomplished.

## Methods

### Field

Field work was conducted in 17 states of Mexico, including in 235 localities in: Chiapas (16), Chihuahua (2), Distrito Federal (22), Durango (1), Guanajuato (2) Guerrero (8), Hidalgo (64) State of México (51), Michoacán (5), Oaxaca (16), Puebla (17), Querétaro (1), Quintana Roo (2), Tlaxcala (15), Veracruz (10), Yucatán (1) and Zacatecas (2).

Emic-type interviews with an ethicist focus took place [9]; meetings were in rural areas, small towns, villages and cities. Their goal was to investigate the tracking, gathering, fixing and commercialization.

For collected, we use aerial nets, paint-brushes, knives or "machetes" and some by hand.

The larvae and pupal stages were placed in 70% alcohol solution or on dry ice if they were intended for chemical analysis. Adults were placed in potassium cyanide with plaster and then put in glassed paper envelopes labeled with the data.

### Laboratory

For identification, adults were placed in a humid camera and mounted; after labeled, identified and catalogued. Forward were placed in the National Collection of Edible Insects of Mexico kept in the Institute of Biology, UNAM. The immature stages were placed in Khale Liquid for preservation. For identification, keys were used [10-17]. Our determinations were ratified by several specialists. With this information, the corresponding tables were elaborated.

The identification of hosts and the ecosystems was accomplished using different sources: De Vries [18], Martínez [19] and Rzedowski [20].

## Results and Discussion

### Diversity and ethnicity

We identified 67 species of Lepidoptera as being eaten in Mexico, in Table 1 shows family, subfamily, scientific name, places of consumption, developmental stage or stages consumed, common name, principal ethnia that use them as food, hosts, and principal ecosystems where they were localized.

**Table 1:** TAXONOMY

FAMILY HEPIALIDAE	
1.- ***Phassus trajesa ***Schaus.	
**Places**:	Argovia, Independencia, Ixtapa, **Chiapas**.
**Edible stage**:	larvae.
**Common names**:	gusanillo (Esp), gusano tindáco (Zap), gusano yutu lolo (Mix).
**Ethnos**:	Maya, tzotzil, tzeltal, chol, lacandon, tojolabal.
**Host**:	*Buddleia americana *L., *Senecio salignus D.C.*
**Ecosystems**.	Pine-oak forest, Tropical decidous forest, Arid tropical scrub, Cloud forest, Rain forest.
2.- ***Phassus triangularis ***Edwards. 1885	
**Places**:	Santa Ana Tlacotenco, San Pablo Oztotepec, San Salvador Cuahtenco, San Pedro Atocpan, San Bartolo Xicomulco, San Antonio Tecomitl, Milpa Alta, **DF**. San Rafael, Pueblo Nuevo, Amanalco de Becerra, Tenancingo, Tequixquiac, Valle de Bravo, **(Mex)**. Yosotato, Coatzospan, Jamiltepec, **Oaxaca**. Necoxtla, Zongolica, **Veracruz**.
**Edible stage**:	larvae.
**Common names**:	Gusanillo (Esp), gusano rayado (Esp), gusano grande (Esp), nduyacacitl (Mix), gusano gordo de la jarilla (Esp), chiáhuitl (Ntl).
**Ethnos**:	Yutoazteca, Nàhuatl, Otomí, Otopame, Mazahua, Matlazinca, Zapoteco, Mixteco, Mixe, Popoluca, Chatinos, Chinantecos, Mazatecos, Zoques, Triques, Huave, Totonaca, Huasteco.
**Host**:	*Buddleia parviflora *H.B.K., *Senecio salignus *D.C.
**Ecosystems**:	Arid tropical scrub, Rain forest.

3.- ***Phassus *sp**.	
**Places**:	San Bartolo Xicomulco, San Pedro Atocpan, Milpa Alta, **DF**. Jilotepec, Cuautitlán de Romero Rubio, Villa del Carbón, San Rafael, Pueblo Nuevo **Mex**. Santo Tomás, Tecomulco, Carpinteros, Atlapexco, Durango, Huasca, Hueyapán, **Hidalgo**. Cañada, Coatzospan, Jamiltepec, San Juan Coatzalapan, Yosotato **Oaxaca**. Santiago Yancuitlalpan, Cuetzalan, Alchichica, **Puebla**. Felipe Carrillo Puerto, **Quintana Roo**. San Pablo del Monte, Xicoténcatl, Xaltocan, Tetla, **Tlaxcala**. Chocamán, **Veracruz**.
**Edible stage**:	larvae.
**Common name**:	gusanillo (Esp), gusano del aile (Esp), gusano del Tepozán (Xoc), calpulocuillin (Ntl).
**Ethnos**:	Yutoazteca, Nàhuatl, Otomí, Otopame, Mazahua, Matlazinca, Mixe, Popoluca, Chatinos, Chinantecos, Mazatecos, Zoques, Triques, Huave.
**Host**:	*Senecio salignus *D.C.
**Ecosystems**.	Arid tropical scrub, Rain forest.

**FAMILY COSSIDAE**	
4.- **Subfamily**: Chilecomadiinae	
***Comadia redtenbacheri ***Hammerschmidt 1848 (Figure 3)	
**Places**:	San Pedro Atocpan, San Salvador Cuahtenco, San Jerónimo Miacatlán, Santa Ana Tlacotenco, San Bartolo Xicomulco, San Lorenzo Tlacoyucan, San Agustín Ohtenco, San Pablo Oztotepec, San Antonio Tecomitl, San Francisco Tecoxpa, San Juan Tepenahuac, Milpa Alta, **DF**. San Bartolo Morelos, Santiago Tianguistenco, Almoloya de Juárez, Villa Nicolás Romero, Oxtotipac, Lomas de Guadalupe, Cuautitlán Izcalli, Cuautitlán de Romero Rubio, San Juan Zitlaltepetl, Villa del Carbón, Santiago Tilapa, Almoloya del Río, Atlacomulco, Ixtlahuaca, Jalatlaco, Zumpango, Ozumba, San Pablo Jalalpan, Toluca, **Mex**. Valle de Santiago, **Guanajuato**. Venta de Guadalupe, Pachuquilla, Pinalito, Pozuelos, San Miguel Regla, Tlaxcoapan, Tulancalco, Trancas, Molango, Tepetitlán. Tulancingo, Zimapán, Cieneguillas, Durango, El Cajón, Ismolintla, El Dexthi, San Juanico, Ixmiquilpan, Pachuca, Tula de Allende, Jacala, San Sebastián Jonacapa, Tinaco, Tezontepec, Santa Ana Bertha, Chapantongo, Atotonilco de Tula, Maravillas, Hueyapán, Singuilucan, Santo Tomás, Cuautepec, Texcaltepec, Chilcuautla, Xochitlán, Venustiano Carranza, Actopan, Valle del Mezquital, Ajacuba, Apan, Atotonilco el Grande, Huichapán, Mixquihuala, San Nicolás Atexcoco, San Antonio Sabanillas, Singuilucan, **Hidalgo**. San Pedro Tarímbaro, **Michoacán**. Vigastepec, Santiago Apoala Santa María Nduayaco, Tlacolula, Ocotlán, **Oaxaca**. Tehuacán, Chapulco, Acatlán de Osorio, **Puebla**. San Juan del Río, **Querétaro**. Calpulalpan, Cuapixtla, Huamantla, Ixtacuixtla, San Pablo del Monte, Tetla, Totolac, Xicoténcatl, Apizaco, San Pablo Matamoros, **Tlaxcala**. Jalpa, **Zacatecas**, Perote, Naolinco, **Veracruz**.
**Edible stage**:	larvae.
Common name:	gusano rojo de maguey (Esp), chilocuiles (Ntl), gusanitos de la sal (Esp), chicuil (Maz), tecol (Oto), chilocuilen (Ntl).
**Ethnos**:	Yutoazteca, Náhuatl, Otomí, Otopame, Mazahua, Matlazinca, Tarasco, Totonaco, Maya, Huasteco.
**Host**:	*Agave atrovirens *Karw, *A. salmiana*, Otto ex Salm, *A. mapisaga *Trel.
**Ecosystems**:	Desert.

**FAMILY PYRALIDAE**	
5.- **Subfamily **Pyraustinae	
***Laniifera cyclades ***Druce 1895	
**Places**:	San AntonioTecomitl, San Francisco Tecoxpa, San Agustín Ohtenco, Milpa Alta, Tlaltenco, **DF**. San Pablo Jalalpan, Oxtotipac, Cerro de las Promesas, Acuitlapilco, Canalejas, Los Reyes, San Juan Teotihuacan, **Mex**. Ajacuba, Cardonal, Chapantongo, Cuautepec, Texcaltepec, Valle del Mezquital, Hueyapan, Tulancalco, Santo Tomás, Tezontepec, Maravillas, Actopan, Alfajayucan, El Dexthi, San Juanico, Ixmiquilpan, **Hidalgo**. Tetla, **Tlaxcala**.
**Edible stage**:	larvae.
Common names:	**gusano del nopal (Esp), citlacuilli (Oto), citlalin (Ntl)**.
**Ethnos**:	Yutoazteca, Náhuatl, Otomí, Otopame, Mazahua, Matlazinca.
**Host**:	*Opuntia *spp.
**Ecosystems**:	Desert.

**FAMILY SESIIDAE**	
**6.- Subfamily **Sesiinae	
***Synanthedon cardinalis ***Dampf.	
**Places**:	MesetaTarasca, **Michoacán**
**Edible stage**:	larvae.
**Common names**:	gusanos cremosos, gusano blanco, mantecoso (Esp), cuillin (Ntl).
**Ethnos**:	Tarasco, Náhuatl, Otomí.
**Host**:	*Pinus *spp.
**Ecosystems**:	Pine-oak forest

**FAMILY CASTNIIDAE**	
7.- **Subamily **Castniinae	
***Castnia synpalamides chelone ***(Hopffer 1856) (Figure 4)	
**Places**:	San Sebastián Jonacapá, Texcaltepec, Mixquihuala, Valle del Mezquital, Venustiano Carranza, Xochitlán, Maravillas, Santa Ana Bertha, Tula de Allende, Zimapán, Pachuca, Singuilucán, Tezontepec, Atotonilco, Tula, Cuautepec, Chapantongo, Chilcuautla, Santo Tomás, Golondrinas, El Dexthi, San Juanico, Ixmiquilpan, Trancas, Ismolintla, Cantayame, **Hidalgo**.
**'Edible stage**:	larvae.
**Common names**.	Gusano del junquillo (Esp), gusanito (Esp), tzic (Oto), papalotillo (Esp).
**Ethnos**:	Náhuatl, Otomí.
**Host**:	*Agave striata. *Zucc.
**Ecosystems**.	Desert.

**FAMILY GEOMETRIDAE**	
8.- **Subfamily**: Ennominae	
***Acronyctodes mexicanaria ***(Walker 1860)	
**Places**:	Topilejo, Santa Ana Tlacotenco, San Lorenzo Tlacoyucan, San Juan Tepenahuac, San Pedro Actopan, Milpa Alta, **DF**.
**Edible stage**:	larvae and pupae.
**Common names**:	Temictli (Oto), Tetatamachiuhqui (Ntl).
**Ethnos**:	Yutoazteca, Náhuatl, Otomí.
**Host**:	*Budleia *spp.
**Ecosystems**.	Savanna, arid tropical scrub, Oak-Forest.

9.- **Subfamily**: Ennominae	
***Panthera pardalaria ***Hübner 1823	
**Places**:	San Simón Tlatlahuilpa **Tlaxcala**, Torres del Potrero **DF**.
**Edible stage**:	larvae
**Common names**:	Huitzitsi (Oto).
**Ethnos**:	Náhuatl, Otomí, Yutoazteca.
**Host**:	Family Graminae
**Ecosystems**:	Cultures of graminae

**FAMILY HESPERIIDAE (Figura 5)**	
10.- **Subfamily Megathyminae**	
***Aegiale hesperiaris ***(Walker 1856) (Figure 5)	
**Places**:	San Pedro Atocpan, San Salvador Cuahtenco, San Jerónimo Miacatlán, Santa Ana Tlacotenco, San Bartolo Xicomulco, San Lorenzo Tlacoyucan, San Agustín Ohtenco, San Pablo Oztotepec, San Antonio Tecomitl, San Francisco Tecoxpa, San Juan Tepenahuac, Milpa Alta **DF**. San Juan Zitlaltépetl, Santa María Jajalpan, San Bartolo Morelos, Huixquilucán, Lomas de Guadalupe, Cuautitlán Izcalli, Cuautitlán de Romero Rubio, Aculco, Almoloya de Juárez, Santiago Tianguistenco, Almoloya del Río, Atlacomulco, Ixtlahuaca, Jalatlaco, Jilotepec, Zumpango, Los Reyes, Ozumba, San Pablo Jalalpan, Toluca, Villa del Carbón, Villa Nicolás Romero, Otumba, Arroyo Zarco, Santiago Tilapa, El Oro, Aguatepec, San Pedro de los Baños, San Mateo, **Mex**. Guanajuato, **Guanajuato**. Santo Tomás, Huichapán, Chilcuatla, San Nicolás Atexcoco, Maravillas, Zimapán, Cuautepec, Jacalá, Pinalito, Ixmiquilpan, Pozuelos, Cieneguillas, Ajacuba, Apan, Atotonilco el Grande, Atotonilco de Tula, Texcaltepec, Tlaxcoapan, Tulancalco, Valle del Mezquital, Xochitlán, Tulancingo, Durango, el Cajón, Pachuquilla, San Miguel Regla, Metztitlán Mixquihuala, Molango, Pachuca, Singuilucan, Tula de Allende, Trancas, Ismolintla, Venustiano Carranza, Venta de Guadalupe, San Sebastián Jonacapa, Tinaco, Santa Ana Bertha, Tezontepec, Chapantongo, Tepetitlán, El Sauce, Ixtaltepec, Alfajayucan, El Dexthi, San Juanico, Ixmiquilpan, **Hidalgo**. Tlalpujahua, San Pedro Tarimbaro **Michoacán**, Santa María Nduayaco, Santiago Apoala, **Oaxaca**, Ciudad Serdán, Acatlán de Osorio, **Puebla**. San Juan del Río, **Querétaro**, Calpulalpan, Cuapixtla, Huamantla, Ixtacuixtla, Nativitas, San Pablo del Monte, Tetla, Totolac, Xicoténcatl, Mariano Matamoros, **Tlaxcala**. Perote, Naolinco, **Veracruz**. Fresnillo, **Zacatecas**.
**Edible stage**:	larvae.
**Common name**:	gusano blanco del maguey (Esp), gusanito del maguey (Esp), meocuiles (Ntl), meocuilines (Ntl), ticoco andabi (Mix), zat (Zap), yabi (My), guinches (Maz), Nnchaama (Tar), Chucugame (Mat), huitzipapalotl (Ntl), papálotl (Ntl).
**Ethnos**:	Yutoazteca, Náhuatl, Otomí, Otopame, Mazahua, Matlazinca, Tarasco, Zapoteco, Mixteco, Totonaco, Huasteco, Maya.
**Host**:	*Agave atrovirens *Karw., *A. salmiana*, Otto ex Salm, *A. mapisaga *Trel, *A. lehmanni. *Jacobi, *A. maximiliana, Baker. A. americana.*
**Ecosystems**.	Desert, pine-oak forest.

11.- **Subfamily Pyrginae**	
***Achlyodes pallida ****(*Felder, 1869)	
**Places**.	San Pablo Huixtepec, **Oaxaca**, Tenejapa, **Chiapas**
**Edible stage**:	Larvae
**Common names**:	chiáhuitl (Mix), saltadora (Esp), papalotl (Ntl).
**Ethnos**:	Zapoteco, Mixteco, Mixe, Populaca, Chatino, Chinanteco, Mazateco, Zoque, Trique, Huave, Tojolabal, Maya, Tzotzil, Tzeltal, Chol, Lacandón.
**Host**:	*Citrus aurantium *L., *C. sinensis*
**Ecosystems**:	Cultures of lucerne and maize.

**FAMILY PAPILIONIDAE**.	
**12.- Subfamily **Papilioninae	
***Protographium philolaus philolaus ***(Boisduval, 1836) (Figure 6)	
**Places**:	Caezim, **Yucatán**.
**Edible stage**:	larvae.
**Common name**:	Tlilizic (My).
**Ethnos**:	Maya.
**Host**:	*Annona cherimola*, *A. diversifolia*, *A. purpurea*, *A. reticulata*; *Desmopsis bibracteata *and *Sapranthus *spp.
**Ecosystems**.	Tropical decidous forest.

13.- **Subfamily **Papilioninae	
***Pterourus multicaudata multicaudata ***(Kirby, 1884)	
**Places**:	Santiago Tezontlale, **Hidalgo**.
**Edible stage**:	adult.
**Common name**:	mariposa de colores (Esp), xochiquetzal (Ntl).
**Ethnos**:	Náhuatl, Otomí.
**Host**:	*Fraxinus *sp., *Prunus persica *L., P. *serotina capuli*.
**Ecosystems**.	Decidous forest, Oak forest

**FAMILY PIERIDAE**	
14.- **Subamily **Coliadinae	
***Phoebis agarithe agarithe ***(Boisduval) 1836 (Figure 7)	
**Places**:	Caezim, **Yucatán**
**Edible stage**:	larvae.
**Common names**:	gusano pinto (Esp), pintillo (Esp), clac (My), xicalpapálotl (Ntl).
**Ethnos**:	Maya.
**Host**:	*Cassia tomentosa *L.*, Inga *sp.
**Ecosystems**:	Tropical decidous forest

15.- **Subfamily **Coliadinae	
***Phoebis philea philea ***(Linnaeus 1763)	
**Places**:	Celaya, Irapuato, Guanajuato
**Edible stage**:	larvae.
**Common name**:	Ocuil (Ntl).
**Ethnos**:	Otomi, Tarasco.
**Host**:	*Casia tomentosa *L.; *Senna *spp.
**Ecosystems**.	"Acahual"

16.- **Subfamily **Coliadinae	
***Phoebis sennae marcellina ***(Cramer 1779)	
**Places**:	San Juan Tezompa, Villa Guerrero, **Mex**.
**Edible stage**:	larvae
**Common names**:	Tlaxic (Oto), Papalotli (Ntl), Tzauhqui (Maz).
**Ethnos**:	Otopame, Mazahua, Matlazinca.
**Host**:	*Cassia *sp.; *Senna*, *Inga*
**Ecosystems**:	"Acahual"

17.- **Subfamily **Coliadinae	
***Eurema salome jamapa ***(Reakirt 1866)	
**Places**:	Tempoal de Sánchez, **Veracruz**.
**Edible stage**:	larvae
**Common names**:	Papalotl (Ntl)
**Ethnos**:	Totonaco, Huasteco.
**Host**:	*Picramnia sp*, *Diphysa robinoides *Benth
**Ecosystems**:	"Acahual"

18.- **Subfamily **Pierinae	
***Eucheira socialis socialis ***(Westwood 1834) (Figure 8).	
**Places**:	San Cristóbal de las Casas, **Chiapas**. Caborachi y sudeste de **Chihuahua**. San Antonio Tecomitl, San Francisco Tecoxpa, San Mateo, San Lorenzo Tlacoandula, San Agustín Ohtenco, Santa Ana Tlacotengo, San Jerónimo Miacatlán, Milpa Alta, Tlaltenco, Topilejo, **DF**. La Michilía, **Durango**, Donato Guerra, Villa Victoria, Cerro de las Promesas, Oxtotipac, San Pablo Jalalpan, Valle de Bravo, Villa de Allende, **Mex**. Chacoalcingo, **Guerrero**. Santo Tomás, Valle del Mezquital, Atlapexco, Huasca, Durango, Tecocomulco, Actopán, Maravillas, Tezontepec, **Hidalgo**. Tlalpujahua, Cerro del Gallo, San Pedro Tarímbaro, **Michoacán**, Nochixtlán, Santa María Nduayaco, Santa María de la Asunción, Tlaxiaco, **Oaxaca**. Ciudad Serdán, Chignahupan, Tetela de Ocampo, **Puebla**. Tetla, **Tlaxcala**. Orizaba, **Veracruz**.
**Edible stage**:	larvae.
**Common name**:	mariposa del madroño (Esp), gusano del madroño (Esp), gusano verde de la mixteca (Esp), Nnchaama (Tar).
**Ethnos**:	Maya, Tzotzil, Tzeltal, Chol, Lacandon, Tojolabal Tarahumara, Yutoazteca, Náhuatl, Otomí, Tepehuano, Otopame, Mazahua, Matlazinca, Tlapaneco, Amuzgo, Tarasco, Zapoteco, Mixteco, Mixe, Popoluca, Chatino, Chinanteco, Mazateco, Zoque, Trique, Huave, Totonaco, Huasteco.
**Host**:	*Arbutus xalapensis *H.B.K.*. A. arizonica*, *A. glandulosa *and *A. macrophylla*
**Ecosystems**:	Pine-Oak forest, Arid tropical scrub.

19.- Subfamily Pierinae	
***Eucheria socialis westwoodi ***(Beutelspacher 1984).	
**Places**:	La Michilía, **Durango**.
**Edible stage**:	larvae.
**Common name**:	mariposa del madroño (Esp), gusano del madroño (Esp), gusano verde de la mixteca (Esp), Nnchaama (Tar).
**Ethnos**:	Tepehuano, Tarahumara.
**Host**:	*Arbutus *sp.
**Ecosystems**.	Pine-Oak forest, Arid tropical scrub.

20.- **Subfamily **Pierinae	
***Catasticta teutila teutila ***Doubleday 1847 (Figures 9 and 10).	
**Places**:	San Francisco Tlalnepantla, Xochimilco, Santa Ana Tlacotenco Milpa Alta, Topilejo, **DF**. Juchitepec, **Mex**. Santa María Nduayaco, Santiago Apoala, **Oaxaca**.
**Edible stage**:	larvae, pupae.
**Common name**:	Mariposa del tejocote (Esp), Tlilpapálotl (Ntl).
**Ethnos**:	Yutoazteca, Náhuatl, Otomí, Otopame, Mazahua, Matlazinca, Zapoteco, Mixteco, Mixe, Popoluca, Chatino, Chinanteco, Mazateco, Zoque, Trique, Huave.
**Host**:	*Viscum álbum *L. *Phoradendron velutinum *(DC) Nutt.
**Ecosystems**:	Pine-oak forest, Tropical evergreen forest and Tropical decudous forest.

21.- **Subamily **Pierinae	
***Catasticta flisa flisa ***(Herrich-Schäffer 1853)	
**Places**:	San Francisco Tlalnepantla, Xochimilco, Milpa Alta **DF**.
**Edible stage**:	larvae.
**Common name**:	Mariposa del tejocote (Esp), Nixtapapalotl (Ntl)
**Ethnos**:	Yutoazteca, Náhuatl, Otomí.
**Host**:	*Phoradendron velutinum *(DC) Nutt.
**Ecosystems**:	Pine-oak forest, Tropical evergreen forest and thorn forest.

22.- **Subfamily **Pierinae	
***Catasticta nimbice nimbice ***(Boisduval, 1836).	
**Places**:	San Francisco Tlalnepantla, Xochimilco, Milpa Alta, **DF**.
**Edible stage**:	larvae.
**Common name**:	Papalotl (Ntl), Papalotontle (Oto).
**Ethnos**:	Yutoazteca, Náhuatl, Otomí.
**Host**:	*Phoradendron velutinum *(DC) Nutt.
**Ecosystems**:	Pine-oak forest, Tropical evergreen forest and thorn forest.

23.- **Subfamily **Pierinae	
***Pontia protodice ***(Boisduval & Leconte 1829).	
**Places**:	Valle de México.
**Edible stage**:	Larvae.
**Common names**:	Tilpapalotl (Ntl),
**Ethnos**:	Otopame, Mazahua, Matlazinca.
**Host**:	*Brassica oleracea *L.
**Ecosystems**.	Cultures of lucerne, cabbage, and Oak Forest.

24.**- Subfamily Pierinae**	
***Leptophobia aripa elodia ***(Boisduval, 1836)	
**Places**:	Valle de México.
**Edible stage**:	larvae.
**Common names**:	Chiahuitl (Oto)
**Ethnos**:	Otopame, Mazahua, Matlazinca.
**Host**:	*Brassica rapa *L., *Lepidium sativum *L., *Tropaeolum majus *L.
**Ecosystems**:	Cultures of cabbage, cauliflower and broccoli.

25.- **Subfamily **Nymphalinae	
***Vanessa annabella ***(Field 1971)	
**Places**:	Santo Tomás, **Hidalgo**.
**Edible stage**:	larvae and pupae.
**Common name**:	gusano (Esp), Papalotepito (Ntl), Quiloculin (Oto).
**Ethnos**:	Náhuatl, Otomí.
**Host**:	*Malva *sp., *Althaea *rosea L.
**Ecosystems**:	Pine-oak forest, arid tropical scrub.

26.- **Subfamily **Nymphalinae	
***Vanessa virginiensis ***(Drury, 1773)	
**Places**:	Santo Tomás, **Hidalgo**.
**Edible stage**:	larvae and pupae.
**Common name**:	gusano del llano (Esp), cochipilotl (Ntl).
**Ethnos**:	Náhuatl, Otomí.
**Host**:	*Antirrhinum *sp., *Senecio salignus *D.C., *Gnaphalium *sp., *Antennaria *sp., *Anaphalis *sp., *Myosotis *sp.
**Ecosystems**.	Pine-oak forest, arid tropical scrub.

27.- **Subfamily**: Nymphalinae	
***Nymphalis antiopa antiopa ***(Linnaeus, 1758)	
**Places**:	Sierra Nevada **Mex**, **Puebla**
**Edible stage**:	larvae.
**Common name**:	Temictli (Ntl).
**Ethnos**:	Otopame, Mazahua, Matlazinca, Náhuatl, Totonaco.
**Host**:	*Salyx babilonica *L., *Salix *sp., *Betula, Populus, Celtis, Ulmus*
**Ecosystems**:	Rain forest, Tropical decidous forest.

28.- **Subfamily**: Nymphalinae	
*Chlosyne lacinia lacinia *(Geyer, 1837)	
**Places**:	Bethel, **Chiapas**.
**Edible stage**:	larvae.
**Common name**:	Gusanito (Esp).
**Ethnos**:	Maya, Tzotzil, Tzeltal, Chol, Lacandon, Tojolabal.
**Host**:	*Helianthus annus *L., *Xanthium *sp., *Verbesina *sp., *Ambrosia *sp.
**Ecosystems**:	Rain forest, tropical decidous forest.

29.- **Subfamily**: Biblidinae	
***Hamadryas *sp**.	
**Places**:	Chichén Itzá, Yucatán.
**Edible stage**:	larvae.
**Common name**:	
**Ethnos**:	Maya.
**Host**:	*Dalechampia *sp. *Tragia *sp.
**Ecosystems**.	Tropical decidous forest

30.- **Subamily **Satyrinae	
***Pareuptychia metaleuca ***(Boisduval, 1870).	
**Places**:	Zongolica, **Veracruz**, Atlixco, **Puebla**, Tapachula, **Chiapas**, Pochutla, **Oaxaca**.
**Edible stage**:	larvae.
**Common name**:	gusano gordo, tzotlimichi.
**Host**:	*Panicum *sp.
**Ethnos**:	Totonaco, Huasteco, Náhuatl, Yutoazteca, Maya, Tzotzil, Tzeltal, Chol, Lacandon, Tojolabal Zapoteco, Mixteco, Mixe, Popoluca, Chatino, Chinanteco, Mazateco, Zoque, Trique, Huave.
**Ecosystems**:	Rain forest, Tropical decidous forest, Pine-oak-forest, thorn forest.

31.- **Subfamily **Danainae	
***Danaus gilippus thersippus ***(Bates, 1863) (Figure 11).	
**Places**:	Santo Tomás, Tecozautla, **Hidalgo**.
**Edible stage**:	larvae.
**Common name**:	mariposa del tizmo (Esp), mariposa tiznada (Esp), papalotli (Ntl).
**Ethnos**:	Náhuatl, Otomí.
**Host**:	*Asclepias linaria *Cav., *A. curassavica *L., *Vincetoxicum *sp., *Philibertia *sp., *Nerium *sp., *Stapelia *sp.
**Ecosystems**:	Pine-oak forest, Tropical evergreen forest.

32.- **Subfamily **Danainae	
***Danaus plexippus plexippus ***(Linnaeus, 1758).	
**Subspecies**	*monarca *L.
**Places**:	Tenejapa, **Chiapas**. Santo Tomás, Tecozautla, **Hidalgo**. Angangueo, **Michoacán**.
**Edible stage**:	adult
**Common name**:	mariposa monarca (Esp), mariposa voladora (Esp), mariposa viajera (Esp), xicalpapálotl (Ntl).
**Ethnos**:	Maya, Náhuatl, Otomí, Tarasco.
**Host**:	*Asclepias linaria *Cav., *A. curassavica *L.,
**Ecosystems**.	Pine-oak forest, Tropical evergreen forest.

**FAMILY BOMBYCIDAE**	
33.- **Subfamily**: Bombycinae	
***Bombyx mori ***(Linnaeus, 1758).	
**Places**:	Yosotato, **Oaxaca**.
**Edible stage**:	larvae.
**Common name**:	gusano de seda (Esp), sedaocuilin (Ntl), tzauhquiocuilin (Ntl).
**Ethnos**:	Zapoteco, Mixteco, Mixe, Popoluca, Chatino, Chinanteco, Mazateco, Zoque, Trique, Huave.
**Host**:	*Morus rubra var rubra *L.
**Ecosystems**.	Cloud forest, Rain forest.

**FAMILY LASIOCAMPIDAE**	
**34.- Subfamily **Lasiocampinae	
***Eutachyptera psidii ***(Sallé, 1857)	
**Places**:	Laguna Atezca, Molango, **Hidalgo**.
**Edible stage**:	larvae
**Common name**:	Mecta'che (Ntl), tecilli (Oto).
**Ethnos**:	Náhuatl, Otomí.
**Host**:	***Psidiun guajaba *L**.
**Ecosystems**:	Cloud forest

**FAMILY SATURNIIDAE**	
35.**- Subfamily **Arsenurinae	
***Arsenura armida ***(Cramer, 1779) (Figure 12).	
**Places**:	Molango, **Hidalgo**. Jamiltepec, **Oaxaca**. Cuezalán, Santiago Yancuitlalpan, Coatepec de Matamoros, Acatlán de Osorio **Puebla**, Santiago Tuxtla, Los Tuxtlas, el Bajío, Chocamán, Ixcohuapa, **Veracruz**.
**Edible stage**:	larvae.
**Common name**:	Cuecla (Ntl), serpiente de mil cabezas (Esp), culebron (Esp), chonocuile (Mzt), cuetano (Mix), pochocuil (Zap), Zapala (Mx), tilpapálotl (Ntl), Tecocoz (Pop).
**Ethnos**:	Náhuatl, Otomí, Zapoteco, Mixteco, Mixe, Popoluca, Chatino, Chinanteco, Mazateco, Zoque, Trique, Huave, Totonaco, Huasteco.
**Host**:	*Ceiba pentandra *L. (Pochote), *Chorisia *sp. *Heliocarpus appendiculatus *Turcz.
**Ecosystems**:	Tropical decidous forest, Tropical evergreen forest, Pine oak-forest.

36.- **Subfamily **Arserurinae	
***Arsenura polyodonta ***(Jordan, 1911).	
**Places**:	Atzitzihuacán, Atlixco, **Puebla**.
**Edible stage**:	larvae.
**Common name**:	cuecla (Ntl), zats (Tot), cuitlame (Maz), gusano del jonote (Esp).
**Ethnos**:	Náhuatl, Totonaco.
**Host**:	**Malvaceae, Tiliaceae, *Chorisia *sp**.
**Ecosystems**:	**Pine-oak forest**.

37.- **Subfamily **Arserurinae	
***Caio championi ***(Druce, 1886).	
**Places**:	sur de **Veracruz**.
**Edible stage**:	larvae.
**Common names**:	Cuillicuatl (Ntl)
**Ethnos**:	**Nahuatl**, Totonaco, Huasteco.
**Host**:	*Bombacopsis *sp., *Chorisia *sp., *Tilia *sp.
**Ecosystems**.	Tropical decidous forest, Tropical evergreen forest.

38.- **Subfamily **Arserurinae	
***Caio richardsoni ***(Druce, 1890).	
**Places**:	Cahuaré **Chiapas**. Chapantongo **Hidalgo**.
**Edible stage**:	Larvae.
**Common names**:	Guano oscuro (Esp), ocul (Ntl), culli (Oto).
**Ethnos**:	Maya, Tzotzil, Tzeltal, Chol, Lacandon, Tojolabal, Náhuatl, Otomí.
**Host**.	*Chorisia *sp., *Ceiba pentandra *L.
**Ecosystems**:	Mesquite-grassland, Arid tropical scrub, Tropical decidous forest, Tropical evergreen forest.

39.- **Subfamily **Ceratocampinae	
***Eacles aff. ormondei yucatanensis ***(Lemaire, 1988)	
**Places**:	Zongolica, Ixcohuapa V**eracruz**
**Edible stage**:	Larvae
**Common names**:	Tlecocoz (Oto).
**Ethnos**:	Náhuatl, Yutoazteca. Otomí.
**Host**:	*Quercus *sp., *Rhus *sp.
**Ecosystems**:	Cloud forest, Oak-forest.

40.- **Subfamily **Ceratocampinae	
***Eacles *sp**. Hübner	
**Places**:	Puerto Morelos, **Quintana Roo**.
**Edible stage**:	larvae.
**Common name**:	gusanito (Esp), xixicalticon (My).
**Ethnos**:	Maya.
**Host**:	Malvaceae, Melastomataceae.
**Ecosystems**:	Tropical evergreen forest.

41.- **Subfamily **Hemileucinae	
***Hemileuca *sp**. (Walker, 1855)	
**Places**:	Zinacantepec, Mercado de Toluca, Almoloya de Juárez, Calixtlahuaca, Villa Victoria, **Mex**.
**Edible stage**:	larvae.
**Common name**:	zacamiches (Maz).
**Ethnos**:	Otopame, Mazahua, Matlazinca.
**Host**:	*Salix *sp., Fagaceae, Leguminosae, Rosaceae.
**Ecosystems**:	Pine-Oak forest.

42.- **Subfamily **Hemileucinae	
***Hylesia frigida ***Schaus, 1911.	
**Places**:	Navenchauc, Zinacantán, Coapilla, **Chiapas**. Santa María Nduayaco, Santiago Apoala, Asunción Nochixtlán, **Oaxaca**.
**Edible stage**:	larvae.
**Common name**:	Nn-chúm (Tzo), calocuillin (Ntl), caliocuillin (Tze).
**Ethnos**:	Maya, Tzotzil, Tzeltal, Chol, Lacandon, Tojolabal, Zapoteco, Mixteco, Mixe, Popolaca, Chatino, Chinanteco, Mazateco, Zoque, Trique, Huave.
**Host**:	*Pinus *sp., *Bursera *sp.. Anacardiaceae, Lauraceae, Melastomataceae.
**Ecosystems**:	Pine-oak forest, Pine-Forest, Cloud forest, Deciduos Forest.

43.- **Subfamily **Hemileucinae	
***Hylesia coinopus ***Dyar, 1913.	
**Places**: Cahuaré, **Chiapas**.	
**Edible stage**:	larvae.
**Common name**:	mariposa de hilo grande (Esp), ciulicuactl (Tze).
**Ethnos**:	Maya, Tzotzil, Tzeltal, Chol, Lacandon, Tojolabal.
**Host**:	*Pinus *sp., *Bursera *sp., Anacardiaceae, Lauraceae, Melastomataceae.
**Ecosystems**:	Tropical decidous forest.

44.- **Subfamily **Hemileucinae	
***Hylesia *sp**. Hübner	
**Places**:	Santa María Asunción, Tlaxiaco, Santa María Nduayaco, Asunción Nochixtlán, **Oaxaca**.
**Edible stage**:	larvae.
**Common names**:	Cuitlicallli (Mix).
**Ethnos**:	Zapoteco, Mixteco, Mixe, Popolaca, Chatinos, Chinantecos, Mazatecos, Zoques, Triques, Huave.
**Host**:	*Pinus *sp., *Bursera *sp.
**Ecosystems**:	Pine-oak forest, Savannah, Desert, **Palmar**.

45.- **Subfamily **Hemileucinae	
***Paradirphia hoegei ***(Druce, 1886).	
**Places**:	Tehuacán Puebla.
**Edible stage**:	Larvae
**Common name**.	Cuchamac (Ntl)
**Ethnos**:	Nahuatl, Totonaco.
**Host**:	*Platanus lindeniana *Mart et Gall, *Salix chilensis *Mol., *Prunus *sp.*, Robinia *sp.
**Ecosystems**:	Rain forest, Tropical decidous forest, Decidous forest, Oak forest.

46.- **Subfamily **Hemileucinae	
***Paradirphia fumosa ***(Felder, 1874).	
**Places**:	Zapotitlán y regiones circunvecinas en la reserva de la Biosfera Tehuacán. **Puebla**.
**Edible stage**:	larvae.
**Common name**:	cuchamá (Ntl).
**Ethnos**:	Nahuatl, Totonaco.
**Host**:	*Eysenhardtia polystachya*, Ortega (Sarg), *Prosopis laevigata*, *Cercidium praecox*, (R et Pav) Harás, *Acacia constricta *Benth.
**Ecosystems**:	Rain forest, Tropical decidous forest, Decidous forest,Oak forest, Arid tropical scrub.

47.- **Subfamily **Hemileucinae	
***Pseudodirphia mexicana ***(Bouvier, 1924).	
**Places**:	Zongolica, **Veracruz**.
**Edible stage**:	adult.
**Common name**:	Popolocon (Oto).
**Ethnos**:	Nahuatl, Yutoazteca, Otomi.
**Host**:	*Fagus *sp., *Quercus *sp., Ulmáceae, Leguminosae, Rosaceae, *Fraxinus uhdei *(Wenz) Ling.
**Ecosystems**:	Cloud forest, Oak forest.

48.- **Subfamily **Saturniinae	
***Antheraea polyphemus mexicana ****(*Hoffmann, 1942).	
**Places**:	Zongolica, **Veracruz**.
**Edible stage**:	larvae.
**Common names**:	Tzucalli (Oto)
**Ethnos**:	Náhuatl, Yutoazteca.Otomí.
**Host**:	*Quercus *sp. *Juglans *sp., *Tila *sp., *Prunus *sp., *Crataegus *sp., *Salix *sp.
**Ecosystems**:	Oak forest, Decidous forest.

49.- **Subfamily **Saturniinae	
***Actias luna ***(Linnaeus, 1758) (Figure 13).	
**Places**:	Sierra Tarahumara, **Chihuahua**.
**Edible stage**:	larvae.
**Common name**:	gusano manchado (Esp), cola de novia (Esp).
**Ethnos**:	Tarahumara.
**Host**:	*Liquidambar *sp., *Juglans cinerea *L., *Diospyros virginiana *L., *Quercus rubra *L..
**Ecosystems**:	Cloud forest, Pine-Oak forest, Desert.

50.- **Subfamily **Saturniinae	
***Actias truncatipennis *(**Sonthonnax 1899)	
**Places**:	Sierra Tarahumara, **Chihuahua**.
**Edible stage**:	larvae.
**Common name**:	gusano gordo (Esp), papalotli (Ntl).
**Ethnos**:	Tarahumara.
**Host**:	*Liquidambar *sp, Juglandacea**e**
**Ecosystems**:	Cloud forest.

**FAMILY SPHINGIDAE**	
**51.- Subfamily **Macroglossinae	
***Pachylia ficus ***(Linnaeus, 1758)	
**Places**:	Motozintla, **Chiapas**.
**Edible stage**:	larvae.
**Common name**:	gusano (Esp).
**Ethnos**:	Maya, Tzotzil, Tzeltal, Chol, Lacandon, Tojolabal.
**Host**:	*Ficus cookii *Stand.
**Ecosystems**:	Forest of *Poeppigia procera *Presl.

52.- **Subfamily **Sphinginae	
***Cocytius antaeus ***(Cramer, 1777) (Figure 14).	
**Places**:	Cahuaré, **Chiapas**, Tixtla, **Guerrero**.
**Edible stage**:	larvae.
**Common name**:	gusano cornudo (Esp), gusano del cuerno (Esp).
**Ethnos**:	Maya, Tzotzil, Tzeltal, Chol, Lacandon, Tojolabal, Náhuatl, Tlapaneco, Amuzgo.
**Host**:	*Anona *spp.
**Ecosystems**:	Thorn forest.

53.- **Subfamily **Sphinginae	
***Manduca sexta ***(Linnaeus, 1763).	
**Places**:	Cahuaré **Chiapas**, Ixcohuapa, **Veracruz**
**Edible stage**:	larvae, adult.
**Common name**:	gusano del cuerno (Esp).
**Ethnos**:	Maya, Náhuatl, Yutoazteca, Otomí.
**Host**:	*Nicotiana tabacum *L.
**Ecosystems**:	Cloud forest, Oak forest.

54.- **Subfamily **Sphinginae	
***Manduca *sp**.	
**Places**:	Ixcohuapa, **Veracruz**.
**Edible stage**:	larvae, adult.
**Common name**:	gusano grande verde (Esp).
**Ethnos**:	Náhuatl, Yutoazteca, Otomí.
**Host**:	*Nicotiana tabacum *L.
**Ecosystems**:	Cloud forest.

**FAMILY NOCTUIDAE**	
55.- **Subfamily **Calpinaenae	
***Ascalapha odorata ***(Linneaus, 1758) (Figure 15).	
**Places**:	Tuxtla Gutiérrez, Bochil, Frontera, Bethel, Selva Lacandona, **Chiapas**. Coyoacán, **DF**. Tixtla, El Potrero, Zacazonapan, Colotlipa, Mezcantepec, Quechultenango, Chilpancingo, **Guerrero**. Teotitlán del Camino, **Oaxaca**. San Juan Epatlán, Izúcar de Matamoros, Atlixco, Ajalpan, Coatepec de Matamoros, **Puebla**. Puerto Morelos, **Quintana Roo**.
**Edible stage**:	larvae.
**Common name**:	mariposa del muerto (Esp), cuetla (Ntl), cuetlacuahuetl (Ntl), pochocuiles (Oto), cuetano (Mix).
**Ethnos**:	Maya, Tzotzil, Tzeltal, Chol, Lacandon, Tojolabal, Yutoazteca, Náhuatl, Otomí, Tlapaneco, Amuzgo, Zapoteco, Mixteco, Mixe, Popoluca, Chatino, Chinanteco, Mazateco, Zoque, Trique, Huave, Totonaco.
**Host**:	Melastomataceae
**Ecosystems**:	Thorn forest, Tropical evergreen forest, Rain forest, Tropical decidous forest.

56.- **Subfamily**: Calpinaenae	
***Ascalapha agarista ***Cramer, 1777.	
**Places**:	Chilpancingo, **Guerrero**
**Edible stage**:	larvae.
**Common name**:	mariposa de la muerte (Esp), mariposa del muerto (Esp).
**Ethnos**:	Náhuatl, Tlapaneco, Amuzgo.
**Host**:	Melastomataceae.
**Ecosystems**.	Tropical decidous forest

57.- **Subfamily**: Agaristinae	
***Gerra sevorsa ***(Grote, 1882).	
**Places**:	Pedregal de San Ángel, **DF**, San Miguel Regla, **Hidalgo**. Misantla, **Veracruz**.
**Edible stage**:	larvae.
**Common name**:	Gusano del maíz (Esp)
**Ethnos**:	Yutoazteca, Náhuatl, Otomí, Totonaco, Huasteco.
**Host**:	**Unknown**
**Ecosystems**.	Cloud forest, Pine-Oak forest, and Arid tropical scrub.

58.- **Subfamilly **Calpinae	
***Latebraria amphipyroides ***(Guenée, 1852)	
**Places**:	Frontera, Echeverria, Argovia, las Cañitas, Selva Lacandona, Ixtapa, Bethel, Independencia, Frontera, **Chiapas**. San Pedro Atocpan, San Salvador Cuahtenco, San Jerónimo Miacatlán, Santa Ana Tlacotenco, San Bartolo Xicomulco, San Lorenzo Tlacoyucan, San Agustín Ohtenco, San Pablo Oztotepec, San Antonio Tecomitl, San Francisco Tecoxpa, San Juan Tepenahuac, Milpa Alta, **DF**. Huejutla de Reyes, Atlapexco, Durango, Santo Tomás, Xochitlán, Chilcuautla, Romantla, **Hidalgo**, Santa María Nduayaco, Santiago Apoala, Huajuapan de Léon, Yosotato, Puerto Escondido, **Oaxaca**. Izúcar de Matamoros, Tehuitzingo, Santa Inés Ahuatempan, **Puebla**. Orizaba, Ixtapaluka, Chocaman, **Veracruz**.
**Edible stage**:	larvae.
**Common name**:	Cuetla (Ntl), Cuetlmami (Oto), culebra gorda (Esp), culebra cornuda (Esp).
**Ethnos**:	Maya, Tzotzil, Tzeltal, Chol, Lacandon, Tojolabal, Yutoazteca, Náhuatl, Otomí, Totonaco, Huasteco, Mixe, Popoluca, Chatino, Chinanteco, Mazateco, Zoque, Trique, Huave.
**Host**.	*Ipomea intrapilosa *Rose
**Ecosystems**:	Rain forest, Tropical decidous forest, Pine-oak Forest, Arid tropical scrub, Pine-Forest.

59.- **Subfamily **Calpinae	
***Thysania agrippina ***(Cramer, 1776)	
**Places**:	Tenejapa, **Chiapas**.
**Edible stage**:	larvae.
**Common name**:	mariposa águila (Esp), mariposon (Esp), mazacuata (Tzo).
**Ethnos**:	Maya, Tzotzil, Tzeltal, Chol, Lacandón, Tojolabal.
**Host**:	Unknown
**Ecosystems**.	Rain forest, Tropical evergreen forest.

60.- **Subfamily **Heliotinae	
***Helicoverpa zea *(Boddie, 1850)**.	
**Places**:	San Pedro Atocpan, San Salvador Cuahtenco, San Jerónimo Miacatlán, Santa Ana Tlacotenco, San Bartolo Xicomulco, San Lorenzo Tlacoyucan, San Agustín Ohtenco, San Pablo Oztotepec, San Antonio Tecomitl, San Francisco Tecoxpa, San Juan Tepenahuac, Milpa Alta, Pedregal de San Ángel, **DF**. Villa de Allende, Polotitlán, Jilotepec, San Francisco Chimalpa, San José Tezompa, Temamatla, Santiago Tilapa, Tequixquiac, **Mex**. Quechultenango, Mezcantepec, Chilpancingo, **Guerrero**. Atlapexco, Chilcuautla, Valle del Mezquital, Durango, Santo Tomás, Xochitlán, Molango, San Miguel Regla, Tlaxcoapan, El Dexthi, San Juanico Ixmiquilpan, **Hidalgo**. Santa María Nduayaco, Santiago Apoala, **Oaxaca**. Tenancingo, Tetla, **Tlaxcala**, Ixcohuapa, **Veracruz**.
**Edible stage**:	larvae.
**Common name**:	gusano del maíz (Esp), gusano de la milpa (Esp).
**Ethnos**:	Yutoazteca, Náhuatl, Otomí, Otopame, Mazahua, Matlazinca, Zapoteco, Mixteco, Mixe, Popoluca, Chatino, Chinanteco, Mazateco, Zoque, Trique, Huave, Totonaco, Huasteco.
**Host**.	*Zea mays *L.
**Ecosystems**:	Cultures of maize mixed with beans, geen beans and lucerne.

61.- **Subfamily **Xyleninae	
***Spodoptera exigua ***(Hübner, 1808).	
**Places**:	Zapotitlán, Tláhuac, **DF**.
**Edible stage**:	larvae.
**Common name**:	gusano soldado (Esp).
**Ethnos**:	Yutoazteca, Náhuatl, Otomí.
**Host**:	*Zea mays *L.
**Ecosystems**.	Cultures of maize.

62.- **Subfamily **Xyleninae	
***Spodoptera frugiperda ***(Smith, 1797).	
**Places**:	San Pedro Atocpan, San Salvador Cuahtenco, San Jerónimo Miacatlán, Santa Ana Tlacotenco, San Bartolo Xicomulco, San Lorenzo Tlacoyucan, San Agustín Ohtenco, San Pablo Oztotepec, San Antonio Tecomitl, San Francisco Tecoxpa, San Juan Tepenahuac, Milpa Alta, **DF**. Villa de Allende, Polotitlán, San José Tezompa, Santa Anita, Temamatla, Tequixquiac, **Mex**. Tlaxcoapan, Tulancalco, **Hidalgo**. Tetla, **Tlaxcala**.
**Edible stage**:	Larvae.
**Common name**:	gusano elotero (Esp).
**Ethnos**:	Yutoazteca, Náhuatl, Otomí, Otopame, Mazahua, Matlazinca.
**Host**:	*Zea mays *L.
**Ecosystems**.	Cultures of maize.

63.- **Subfamily **Xyleninae	
***Spodoptera *sp**.	
**Places**:	Milpa Alta, **DF**.
**Edible stage**:	larvae.
**Common name**;	gusano soldado (Esp).
**Ethnos**:	Yutoazteca, Náhuatl, Otomí.
**Host**:	*Zea mays *L.
**Ecosystems**:	Culture of maize mixed with beans, greenbeans and lucerne.

**FAMILY ARCTIIDAE**	
64. - **Subfamily**: Arctiinae	
***Pelochyta cervina ***(Edwards, 1884).	
**Places**:	Zongolica, **Veracruz**
**Edible stage**:	adult.
**Common Name**:	
**Ethnos**:	Náhuatl, Yutoazteca, Otomí.
**Host**:	Scientific name unknown, common name "cucharilla real".
**Ecosystems**:	Rain forest and Tropical decidous forest.

65.- **Subfamily**: Arctiinae	
***Elysius superba ****(*Druce, 1884) (Figure 16)	
**Places**:	Zongolica, **Veracruz**.
**Edible stage**:	larvae.
**Common name**:	gusano del palo mulato (Esp).
**Ethnos**:	Náhuatl, Yutoazteca, Otomí.
**Host**:	*Bursera simaruba *Sarg., *Ficus *sp.
**Ecosystems**:	Rain forest and Tropical decidous forest.

66.**- Subfamily **Arctiinae	
***Amastus ochreaceator ****(*Walker, 1865) (Figure 17)	
**Places**:	Zongolica, **Veracruz**.
**Edible stage**:	larvae.
**Common name**:	gusano de los palos (Esp), xicaltetecon (Ntl).
**Ethnos**:	Náhuatl, Yutoazteca, Otomí.
**Host**:	*Inga jinicuil *Schl.
**Ecosystems**.	Rain forest and Tropical decidous forest.

67.**- Subfamily**: Arctiinae	
***Estigmene acrea ****(*Drury, 1773) (Figure 18)	
**Places**:	Zongolica, Veracruz.
**Edible stage**:	adult.
**Common name**:	oruga salina (Esp).
**Ethnos**:	Náhuatl, Yutoazteca, Otomí.
**Host**:	*Phaseolus vulgaris, Mimosa af. pigra *L.
**Ecosystems**.	Rain forest, Tropical decidous forest.

Languages: Español (Esp); Matlazinca (Mat); Maya (My); Mazahua (Maz); Mazateco (Mzt); Mixe (Mx); Mixteco (Mix); Nahuatl (Ntl); Otomi (Oto); Popoluca (Pop); Tarasco (Tar); Totonaco (Tot); Tzetzal (Tze); Tzotzil (Tzo); Xochimilca (Xoc); Zapoteco (Zap)

**Figure 1:**
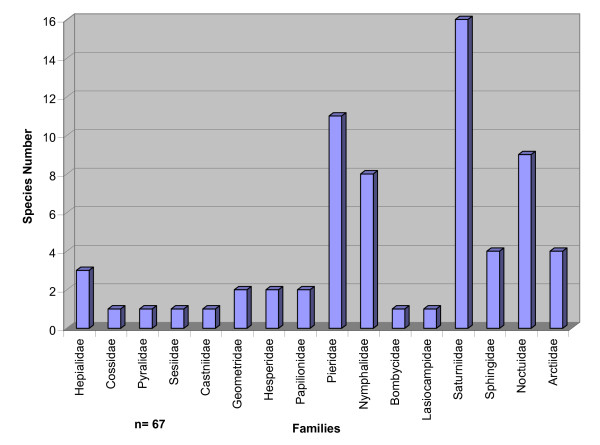
Species Number by families of Edible Lepidoptera, Mexico

The 13 families are in decreasing order of species number: Saturnidae (16), Pieridae (11), Noctuidae (9), Nymphalidae (8), Sphingidae (4), Arctiidae (4), Hepialidae (3), Hesperidae, Papilionidae and Geometridae (2) each one, Cossidae, Pyralidae, Sesiidae, Castniidae, Bombycidae, and Lasiocampidae (1) each one (Table 2, Figure 1).

**Table 2:** Families and species number.

Family	Species	Family	Species	Family	Species
Hepialidae	3	Hesperiidae	2	Saturniidae	16
Cossidae	1	Papilionidae	2	Sphingidae	4
Pyralidae	1	Pieridae	11	Noctuidae	9
Sesiidae	1	Nymphalidae	8	Arctiidae	4
Castniidae	1	Bombycidae	1	Total of species	67
Geometridae	2	Lasiocampidae	1		

**Figure 2:**
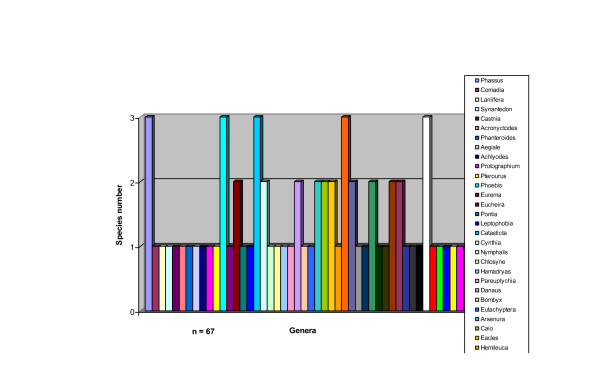
**Species number of edible Lepidoptera in Mexico**.

The species number in each genus is indicated in figure 2. It can be seen that most of the genera have only one species included (68.75%), followed by the bispecific (18.75%) and at the end trispecific genera (12.5%).

The most represented genera were *Phassus, Phoebis, Hylesia, and Spodoptera *(Table 3).

**Table 3:** Genus and species number.

Genus	Species	Genus	Species	Genus	Species.	Genus	Species
*Phassus*	3	*Eucheira*	2	*Caio*	2	*Helicoverpa*	1
*Comadia*	1	*Pontia*	1	*Eacles*	2	*Latebraria*	1
*Laniifera*	1	*Leptophobia*	1	*Hemileuca*	1	*Spodoptera*	3
*Synantedon*	1	*Catasticta*	3	*Hylesia*	3	*Thysania*	1
*Castnia (Synpalamides*)	1	*Cynthia*	2	*Paradirphia*	2	*Pelochyta*	1
*Acronyctode*s	1	*Nymphalis*	1	*Pseudodirphia*	1	*Elisus*	1
*Phanteroides*	1	*Chlosyne*	1	*Antheraea*	1	*Amastus*	1
*Aegiale*	1	*Hamadryas*	1	*Actias*	2	*Estigmene*	1
*Achlyodes*	1	*Pareuptychia*	1	*Pachylia*	1	Total of species	67
*Protoghraphium*	1	*Danaus*	2	*Cocytius*	1		
*Pterourus*	1	*Bombyx*	1	*Manduca*	2		
*Phoebis*	3	*Eutachyptera*	1	*Ascalapha*	2		
*Eurema*	1	*Arsenura*	2	*Gerra*	1		

Lepidoptera are eaten in 85.41% as larvae, 8.33% as larvae and pupae and in 6.25% as adults.

We found 29 ethnic groups that consume Lepidoptera in Mexico: Amuzgo, Chatinos, Chinantecos, Cholos, Huasteco, Huaves, Lacandones, Matlazinca, Maya, Mazahua, Mazatecas, Mixes, Mixtec, Nahuatl, Otomi, Otopame, Popolucas, Tarahumara, Tarascan, Tepehuano, Tlapaneco, Totonaco, Tojolabal, Triques, Tzeltal, Tzotzil, Yutoazteca, Zapotec and Zoques.

### Geographic Distribution

These Lepidoptera species were found in those states of the central, south and southeast regions of the country. The highest number of species (22) was recorded in the eastern part of Veracruz, followed by Hidalgo (17), Distrito Federal (the capital) (16), and Chiapas and Puebla (12 species each). The remaining states, each one had six or fewer edible species.

With regard to the ecosystems [20], these species are attached from the pine oak forest, to the savannah and palmar. The Lepidoptera are also present in several agronomic plants, such as maize, alfalfa, cabbage and cauliflower, depending on the species.

### Anthropolarvifagia of Lepidoptera in the World

Bergier [21] reports 15 species for the world, for America only one species *Hesperiaris *sp., in two countries. Taylor [22] registered 25 species in 12 families. Silow [23] describes 42 species of the genera *Gonimbrasia, Imbrasia, Bunaea, Bunaeopsis, Cirina, Pseudantheraea, Micragone, Olocerina*, and *Melanocera*, 33 of them are eaten in Zambia. It is important to mention that all these authors only did bibliographic research. In contrast, Malaisse and Parent [24] performed long-term field work studying Meridian Shaba area in Republic of Congo and in Zambia reporting 37 species (70% classified) and Latham [25] in Low-Congo, documented 31 species (77% classified). In both studies, the principal families were Attacidae and Notodontidae. Banjo et al. [26] reported six species in Nigeria, four of *Anaphe *genus. Oliveira et al. [27] also reported four species eaten in Angola. We note that six references are books and almost all refer to Africa.

Wen [28] in China presented 66 species, 20 genera and 17 families; 36 species of *Hepialus *genus. Mitsuhashi [29] reported five species in Japan.

Paoletti et al. [30] noted that larvae of Castniidae, Noctuidae and Sphingidae families are consumed in the Amazon area. Our report has 67 species occurring in just a part of the country.

### Rural Nutritional Importance

For rural peasants, the big diversity that Edible Lepidoptera has, besides the good nutritive value achieve (18-57% proteins, 7-77% fats, 0.7-8% minerals, 0.8-25% carbohydrates and 3-29% crude fiber, 231-777 kcal/100 g, [4], and their good flavor that gives their fats, united to the abundance of their populations, conspicuity of their specimens (latest larval stage) that save various important nutrients as proteins and the numerous muscles they posses, combined with their quick preparation (only roasted or boiled), and their innocuity, the easiness to store, make of them an item very searched plus their versability of fix make the Lepidopterans a suitable food, for helping people to have a good health and satisfaction of energy and proteins requirements.

### Marketing and Gastronomy

The trade of Lepidoptera larvae still persist being sold in markets in several areas of the country and even at the capital, as the red and white agave worms. In five forks restaurants of Mexico City. These species are in great demand, in large part due to their exquisite flavor, though the eating of these worms is also an ancestral tradition and a signal of power in diverse sectors of the population. Due to the high demand for these species, some sellers of them have special refrigerators for freezing and storing them. In this way, they can offer and prepare them at high prices after the collecting season.

There are other genera, such as *Phassus*, for which people search laboriously, it has a very similar flavor to chicken, while *Laniifera cyclades "*nopal worm" has a flavor of a fried potato. In the humid-tropical areas, "cuetla" and "cuecla" larvae, corresponding to *Latebraria amphipyroides *and *Arsenura armida *are pickled to give the larvae a flavor similar to herring, while the *Spodoptera *spp. is similar to that of corn (Table 4).

**Table 4:** Genus and Species most consummed in México.

Family	Genus	Species	Family	Genus	Species
Hepialidae	*Phassus*	*Trajesa*	Saturniidae	*Arsenura*	*polyodonta*
	*P.*	*triangularus*		*Caio*	*richardsoni*
	*P.*	sp.		*Hylesia*	*coinopus*
Cossidae	*Comadia*	*redtenbacheri*		*H.*	*frigida*
Castniidae	*Castnia (Synpalamides*)	*chelone*		*H.*	sp.
Pyralidae	*Laniifera*	*cylades*	Noctuidae	*Ascalapha*	*odorata*
Geometridae	*Acronyctodes*	*mexicanaria*		*A.*	*agarista*
Hesperidae	*Aegiale*	*hesperiaris*		*Helicoverpa*	*zea*
Pieridae	*Catasticta*	*teutila teutila*		*Latebraria*	*amphiphyrioides*
	*C.*	*flisa flisa*		*Spodoptera*	*frugiperda*
	*Eucheira*	*socialis socialis*		*S.*	*exigua*
Saturniidae	*Arsenura*	*armida*			

Unfortunately, these organisms are the subject of massive gathering in several of the States of Mexico, where they are profusely eaten. Thus, they could be in danger of extinction, due to the lack of rules regarding their collection, distribution and commercialization [31].

### Cultures and Proto-cultures

In Mexico, some Lepidoptera are raised. *Leptophobia aripa elodia*, *Pieris brassicae *also the silk worm *Bombyx mori *in the States of Oaxaca and San Luis Potosí. Their industrial management is widely known, because of their economical importance in China, Japan, India, France, and Italy.

*Eucheira socialis socialis *the green worm of the Huasteca region widely distributed has larvae that are located inside a secreted silk enclosure of papyraceous consistency. The larvae hang on the branches of *Arbutus xalapensis*, feeding on young leaves [32]. In some parts of it, people make a "protoculture" that maintain on the edges of their house roof. They hang at least three silk enclosures (each bag contains only one sex), if they do that, the protoculture will survive. In the zone of the Oaxaqueña Mixteca, specially in the towns of Santa María Nduayaco and Santiago Apoala, this species disappeared due to the great degree of consumption; this species has since been reintroduced from Durango and Mexico states [33].

Some ethnobiological studies have been conducted on the red and white agave worms [34]. We investigate on their biology, ecology, and ethology, to increase their production by optimization of their culture particularly in Santo Tomàs, Montecillo, and Apan in the State of Hidalgo and in the laboratory [35,36] with this we developed the biotechnology that would allow their culture on a greater scale. In fact, this technology for such cultures can be purchased in the Intelectual Property Direction of the UNAM [37]. Also, studies have been conducted to characterize the development of the larvae of the red agave worm [38].

### Sustainable Management

The management and conservation of the species *Paradirphia fumosa *has been implemented in Mexico at the Biosphere Reserve of Tehuacán-Cuicatlán [39,40].

In this aspect we must also recognize the deep knowledge that indigenous people all over the world have, as they possess 90% of the planet's germplasm [41,42] because they have maintained a high degree of sustainability with the majority of their resources.

### Biomass obtention

These species are recollected by their abundance, because in some ones their recollection could be measure in tons [43] as it happens today with *Ascalapha odorata or Latebraria amphypirioides *stored in big cotton sacs of 50 kg and offered in market day or in a "tianguis" (market of little towns). Other species they could also sell alive while they are inside a little sac that they build in silk form their nests, as it is in gregarious species, *Eucheira socialis *or *Hylesia frigida*. Other species are captured by the use of net as is *Phoebis agarithe*, or the "monarch butterfly" (*Danaus plexipus plexipus*, *D. gilippus thersippus*) or *Pterourus multicaudata multicaudata *where people do not eat the larvae because this makes the heart stop, but adults. In other species they could be found many individuals together inside their hosts as in *Comadia redtenbacheri *or *Laniifera cyclades*, with the help of a hunting knife or even collected the prepupa digging the soil around.

### Preparation

Generally is the larvae that are eaten. They are prepared roasted with salt, and in populations with a higher economic purchase they are fried with oil or lard joining always pepper, salt, in tortillas (maize crepes). They could be boiled and roasted in a "pan" or justly fried with salt and pepper, wormseed leaves. Also, boiled split into longitudinale axis, mixed with oil. Boiled, drained and stuffed with fresh cheese, or it could be with tuna or cooked with eggs, like an omelette. Also in a pie accompanied or mixed with rice, as are the shrimps in the "paella" transfering to rice a very special and good flavour.

They could be preserved in brine, and cutted into small pieces in the same way as used crouttons or bacon.

The flavours are really peculiar and it is dificult to compare with something known, but we can said they varied from light delicious flavours to strong and different unkown flavours.

Peasants qualified them as a very good and nutritious "worms", ¡pure vitamine!, to refer to the quantity of proteins they lodged.

### Trade and Marketing Nets

Many species are traded and sell by fits or sardine cans. Another way to sell them is already boiled in salt water or preserved in brine. Generally, they are not offer in fixed places in trough the market, but street sellers are walking in different corridors of these, asking people to buy them, by example *Comadia redtenbacheri*, *Aegiale hesperiaris*, *Arsenura armida*, *Ascalapha odorata*, and *Latebraria amphipyroides *are sold on the plaza days, market days, in ambulant markets or on roadsides and even in the Mexico City market. They are offered in big plastic boxes or baskets and are measured in tuna or sardine cans, or frequently in "cazuelitas" (little ceramic dishes of different sizes). Some of the recorded edible Lepidoptera thus clearly constitute an important part of the nutrition and economy of the Mexican people [33,34], particularly for the indigenous collectors, middlemen, distributors, salesmen, and restaurant owners. In addition, canned white agave worms are exported to the United States and Canada by the enterprises Clemente Jacques and Elan, S.A., and thus generate foreign income for Mexico. This worm has been sold for $250.00 USD per kilogram (2006), which is ten times more expensive than a fish or beef fillet. The exported cans cost $50.00 Canadian dollars; these cans contain only 5 or 6 larvae of the last or penultimate larval stage.

Other species have also been commercialized, such as "zacamiches", *Hemileuca *sp. at Toluca market, "gusanillo", *Phassus triangularis*, and *P. trajesa *at different markets in the Veracruz State and "cuchama" (*Paradirphia fumosa*) in Tehuacán, Puebla.

A little more than 10% (8 species) of edible Lepidoptera larvae are commercialized, but many more species are sold in the adult stage at very high prices.

Some African species in the larval stage are preserved by pickling and are then exported to European cities. In Paris, France, for example, they are offered in the market of La Rue Moufetard in the Latin neighborhood. These are sold in huge fiber baskets, and can be seen in the street markets in several localities for sale on different days of the week. They are mostly bought by immigrants in those countries [32].

Some examples of Edible Lepidoptera of Mexico are Figures 3, 4,5, 6, 7, 8, 9, 10, 11, 12, 13, 14, 15, 16, 17 and 18.

**Figure 3:**
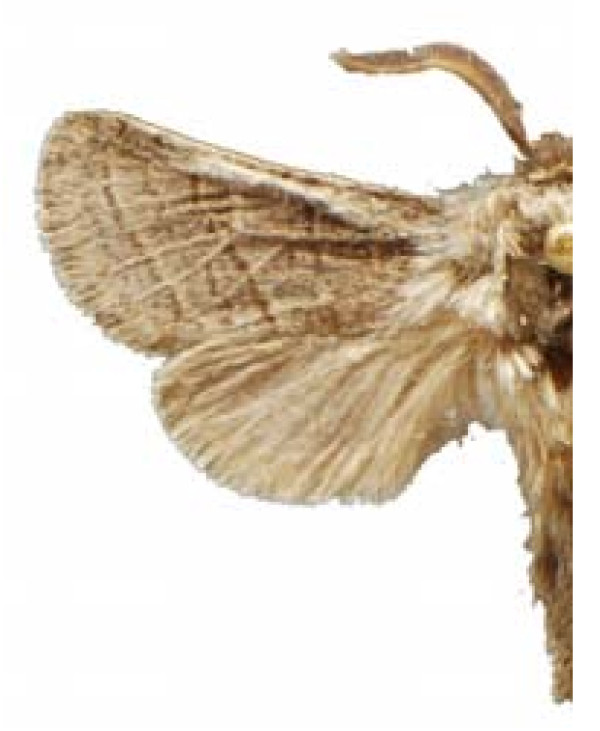
Comadia redtenbacheri (♂)

**Figure 4:**
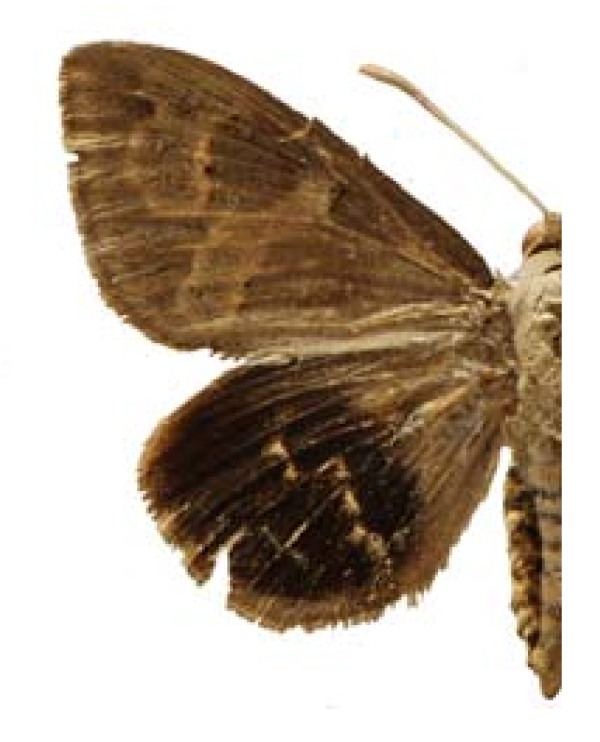
Castnia synpalamides chelone (♂)

**Figure 5:**
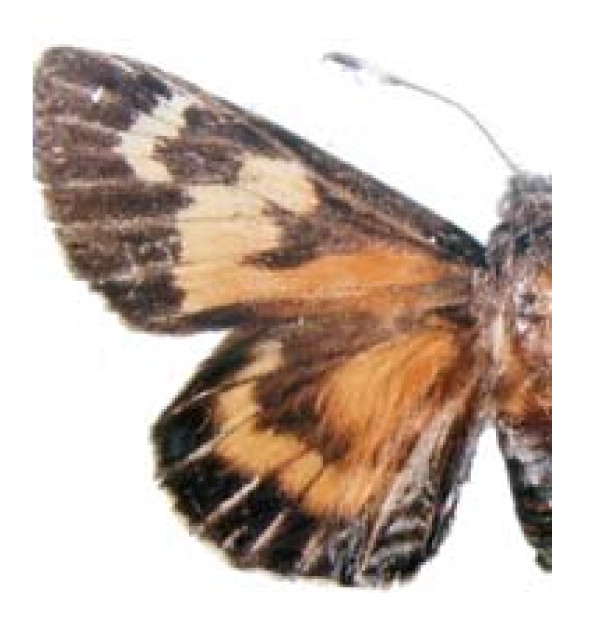
Aegiale hesperiaris

**Figure 6:**
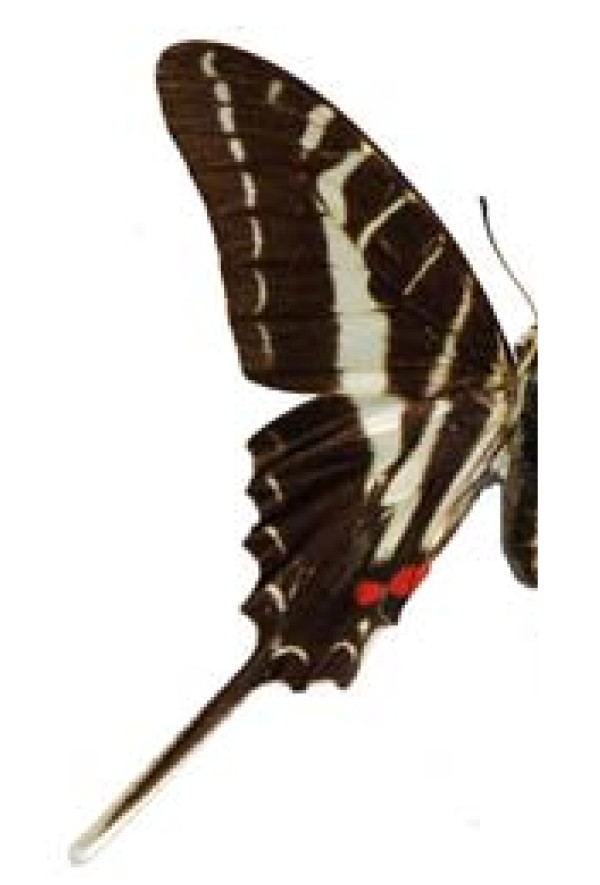
Protographium philolaus philolaus (♂)

**Figure 7:**
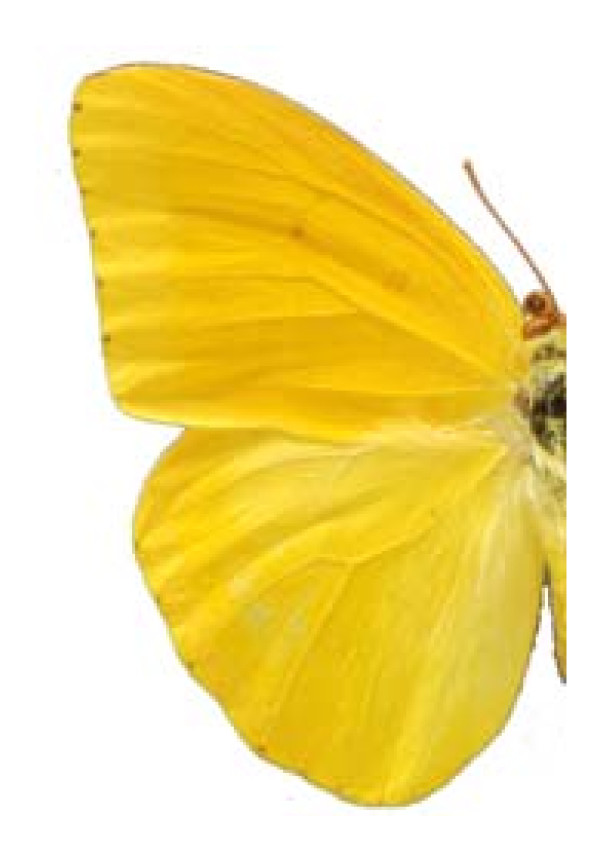
Phoebis agarithe agarithe (♂)

**Figure 8:**
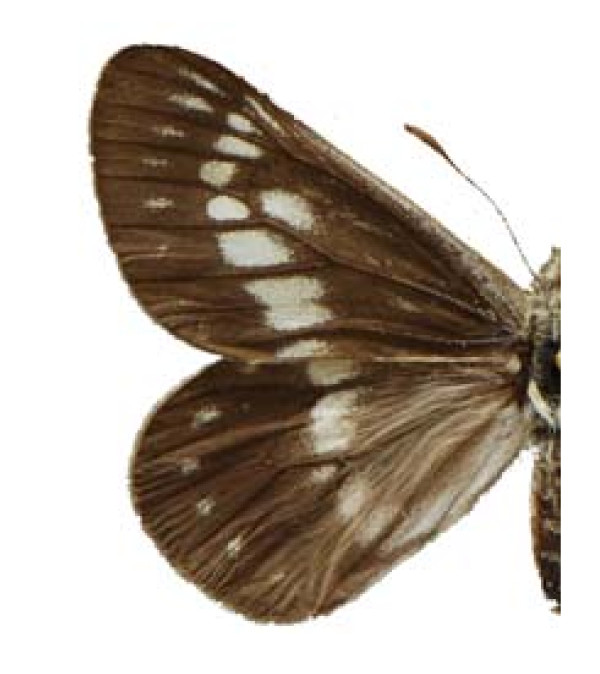
**Eucheira socialis socialis (♀)**.

**Figure 9:**
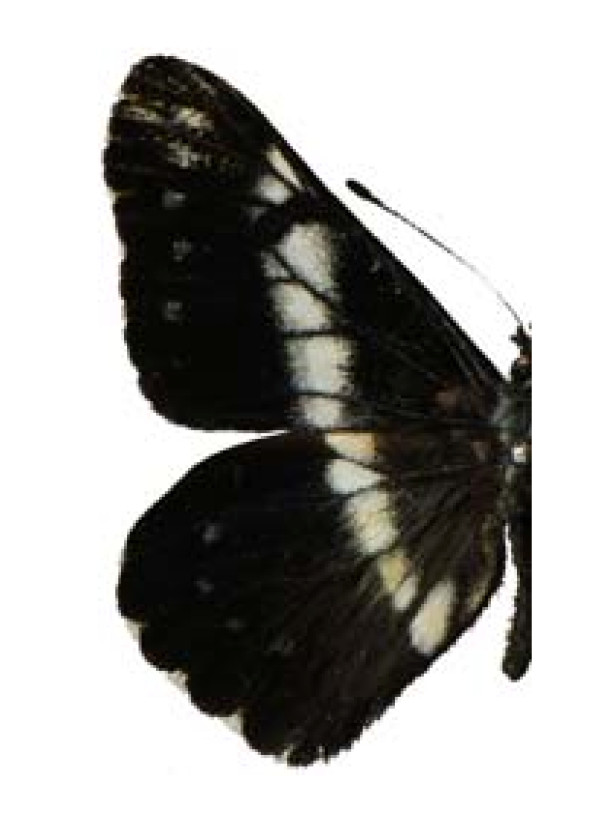
Catasticta teutila teutila (♂)

**Figure 10:**
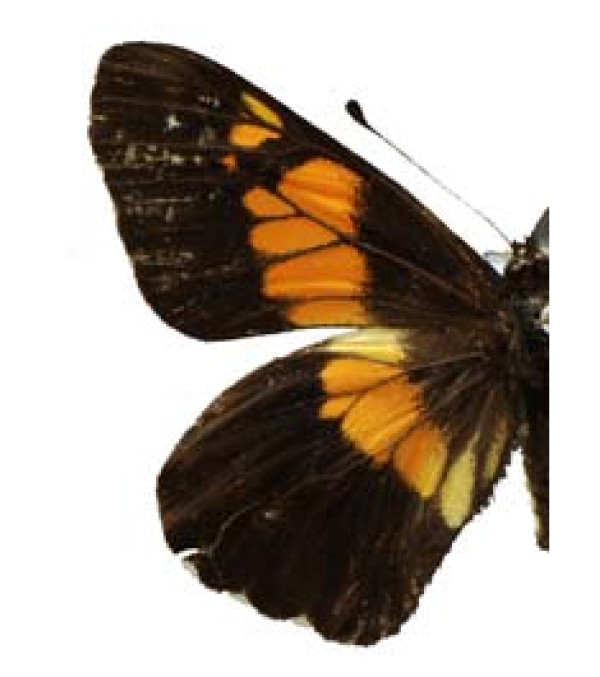
Catasticta teutila teutila (♀)

**Figure 11:**
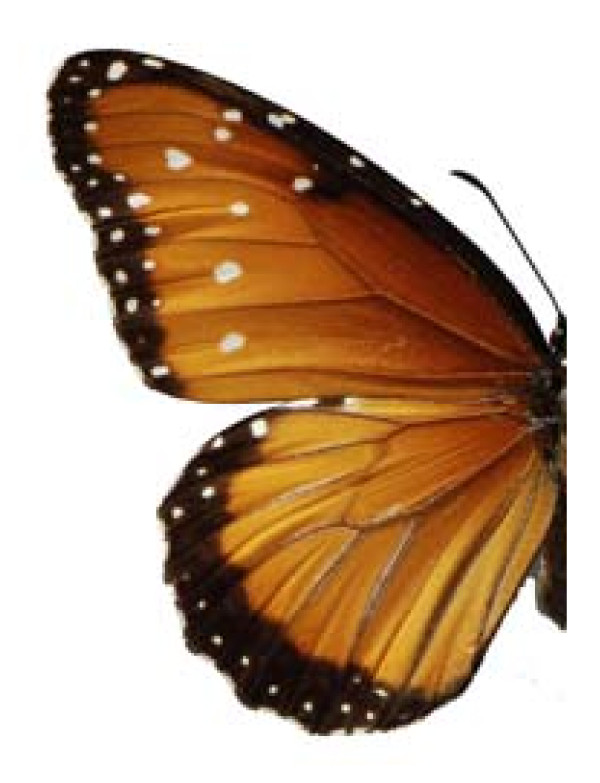
Danaus gilippus thersippus (♀)

**Figure 12:**
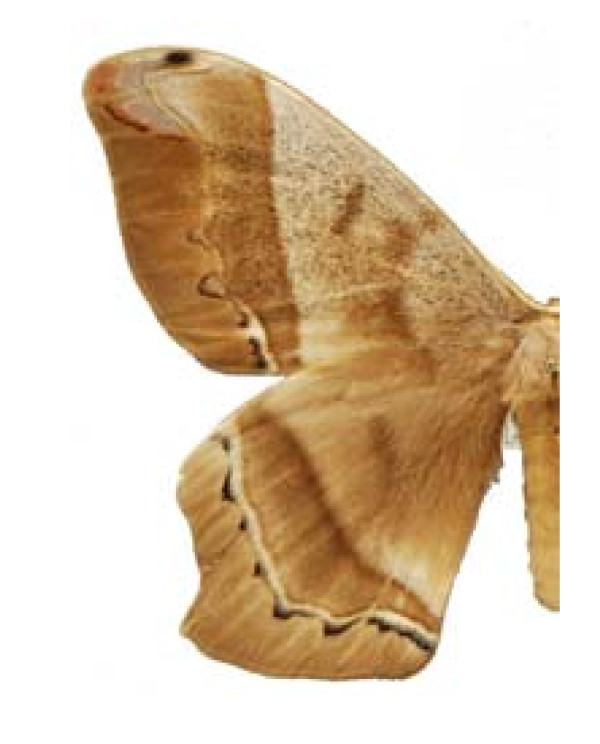
Arsenura armida (♂)

**Figure 13:**
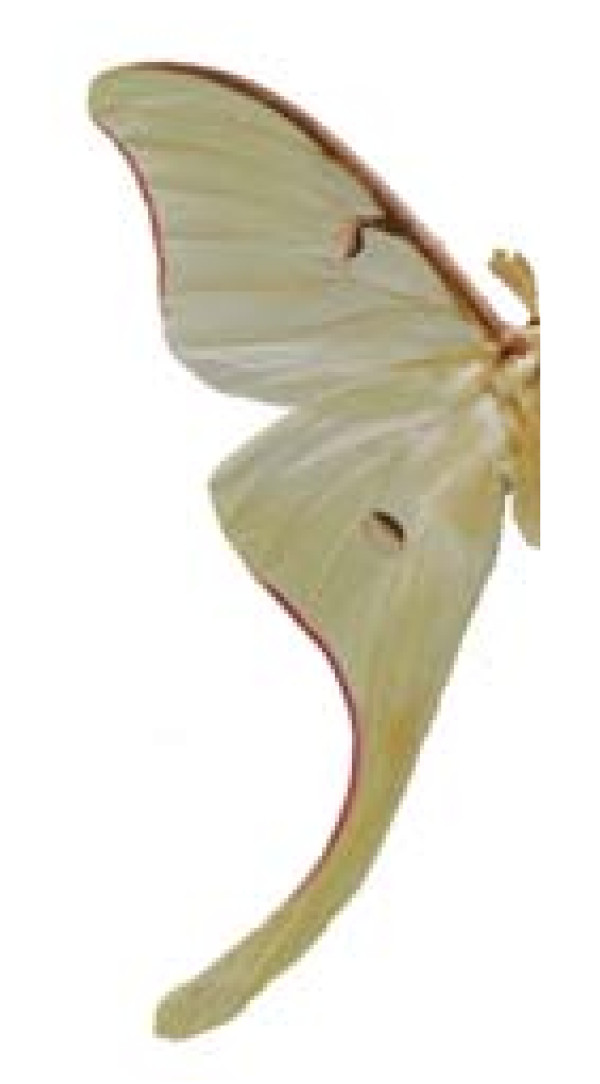
Actias luna (♂)

**Figure 14:**
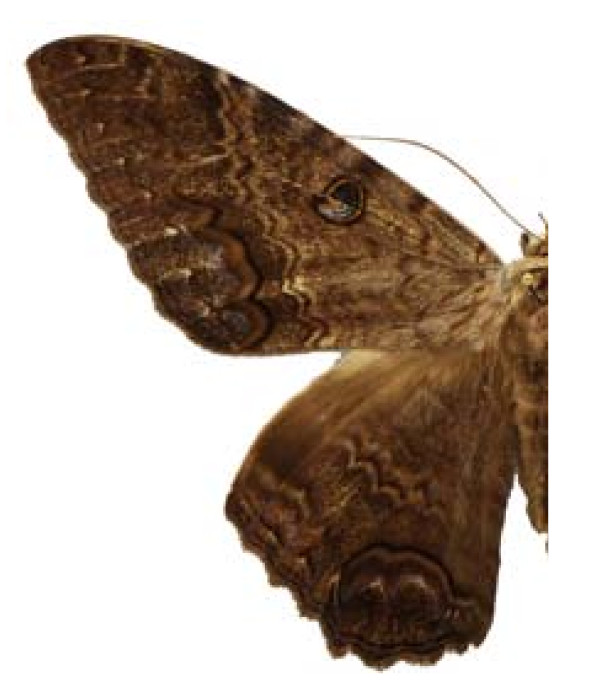
Cocytius antaeus (♂)

**Figure 15:**
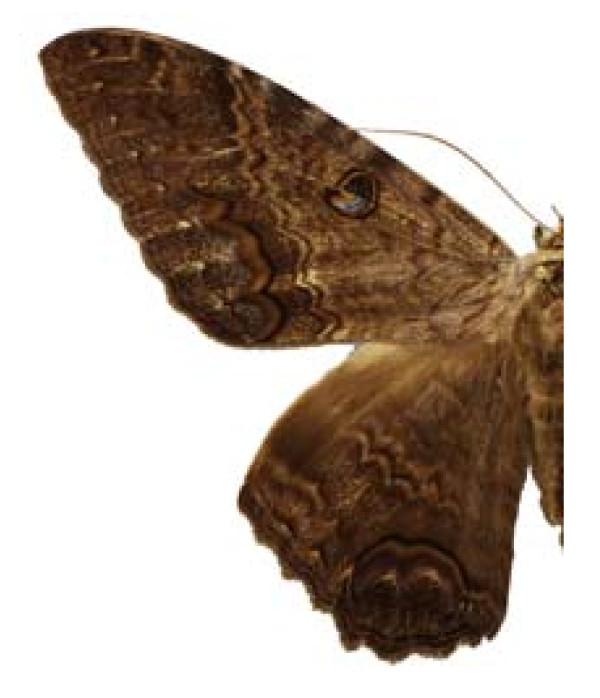
Ascalapha odorata (♂)

**Figure 16:**
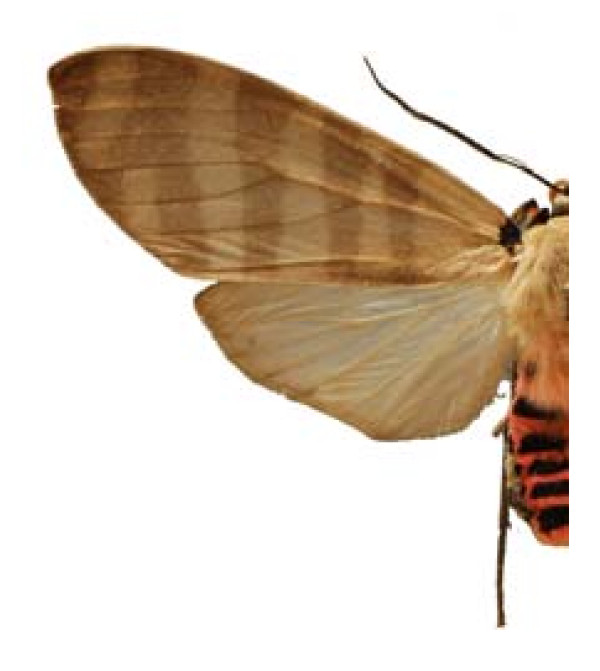
Elysius superba (♂)

**Figure 17:**
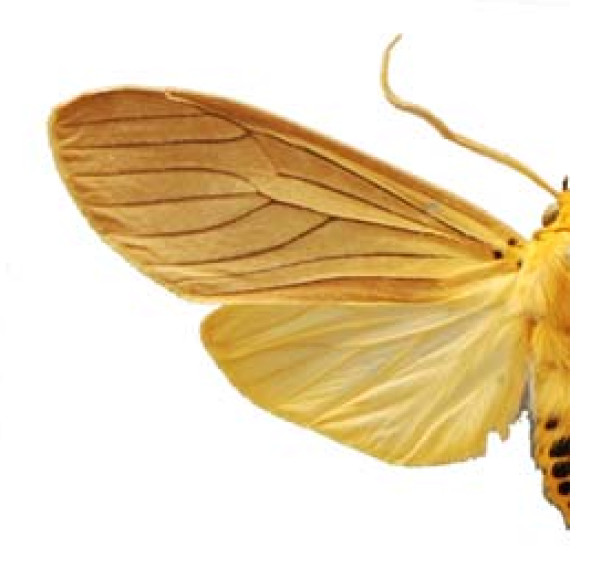
Amastus ochreaceator (♂)

**Figure 18:**
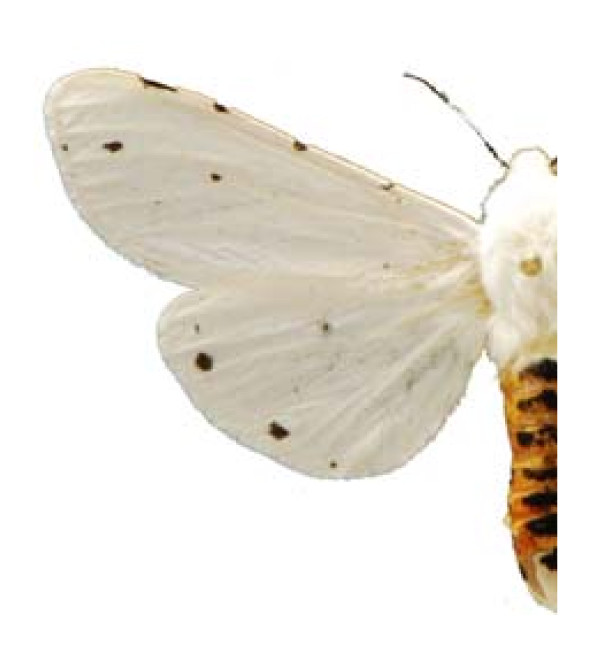
Estigmene acrea (♂)

## Competing interests

The authors declare that they have no competing interests.

## Authors' contributions

All authors read and approved the final manuscript. JRE, Author responsable for the project and publication, writting, editing of the manuscript. JMPM, Collect, preliminary preparation of the manuscript, editing, labels, catalogs, literature review. AIV, Assembly and identification of species of Lepidoptera. ILT, Collect of different species in diverses states of Mexico. HOR, Identification of host plants. VHMC. Writting and formatting the manuscript, references research on internet and all computer work.

## Authors' information

Dra. Julieta Ramos-Elorduy: has the highest position as researcher at the Institute of Biology of the National University of Mexico and professor of postgraduate courses at the Faculty of Science of the same University. She have 104 scientific publications and four books published. 1153 cites of its publications and 1316 on internet. She lead 152 thesis and publish 289 divulgation articles.

M.en C. José Manuel Pino Moreno: Biologist and M.Sc. by the Faculty of Science of the UNAM (National University of Mexico), Academic Technical of the Institute of Biology and Professor of the Faculty of Sciences both of the UNAM. He has published like co-author several articles about antropoentomophagy and medicinal insects and one book.

Adolfo Ibarra Vázquez. Technical Lepidoptera collection of the Institute of Biology of the UNAM.

M en C. Ivonne Landero Torres. Biologist and M.Sc. by the University of Veracruz, Urban Management and Promotion, Professor of the Faculty of Biology, Cordoba. She has published like co-author several articles about anthropoentophagy.

Héctor Oliva-Rivera. Biologist and M.Scy by the University of Veracruz, Plant Taxonomy Professor of the Faculty of Biology, Cordoba.

Biologist Victor Hugo Martínez Camacho by the Faculty of Science of the UNAM (National University of Mexico), he has published like co-author one chapter of book and several articles of edible insects.
